# The UK Antimicrobial Registry (UKAR): an overview of the first 20 months of recruitment

**DOI:** 10.1093/jacamr/dlaf242

**Published:** 2025-12-17

**Authors:** Jacqueline Sneddon, Rebecca Parr, Jay Woods, Ross I R MacDonald, Jonathan A T Sandoe, Ioannis Baltas, R Andrew Seaton, Noha El Sakka, Callum Kaye, Gary J Macfarlane, Gareth T Jones, the UKAR Study Principal Investigators, Ceri Phillips, Yee Pang, Faisal Khan, Clara Tam, Claire Brandish, Ruth McAleer, Stephen Hughes, Justin Cooke, Siân Price, Rakhee Patel, Syed Anas Daud Gilani, Jonathan Urch, Lorraine Cullen, Rhiannon Robinson, Mark Gilchrist, Emily MacNaughton, Amy Lee, Jasmin Islam, Jonathan Sandoe, Danielle Lucy, Anne Neary, Graciela Sluga, Frances Garraghan, Louise Sweeney, Stuart Bond, Hannah Soulsby, Euan Proud, Fiona Thorburn, Noha El Sakka, Andrew Seaton, Simon Dewar, Charis Marwick, Clare Hamson, Joseph Brayson, Annette Clarkson, Helen Chesterfield, Neil Powell, Hazel Parker, Sumita Pai, Nicola Marsh, Andrew Brush, Helena Parsons, Sajeevan Rasanantham, Angel Boulos, Marina Basarab, Julie Harris, Ana Lima Soares, Peter Gayo Munthali, Abigail Jenkins, David Jenkins, Yasmin Elmasry, Racheol Sierra, Karamjit Badyal

**Affiliations:** British Society for Antimicrobial Chemotherapy, Birmingham, UK; Epidemiology Group, University of Aberdeen, Aberdeen, UK; Epidemiology Group, University of Aberdeen, Aberdeen, UK; Epidemiology Group, University of Aberdeen, Aberdeen, UK; British Society for Antimicrobial Chemotherapy, Birmingham, UK; School of Medicine, University of Leeds, and Leeds Teaching Hospitals NHS Trust, Leeds, UK; British Society for Antimicrobial Chemotherapy, Birmingham, UK; Infection, Immunity & Inflammation Department, UCL Institute of Child Health, London, UK; British Society for Antimicrobial Chemotherapy, Birmingham, UK; Department of Medicine, Queen Elizabeth University Hospital, NHS Greater Glasgow and Clyde, UK; Department of Medical Microbiology, NHS Grampian, Aberdeen, UK; Anaesthetics & Critical Care, NHS Grampian, Aberdeen, UK; Epidemiology Group, University of Aberdeen, Aberdeen, UK; Epidemiology Group, University of Aberdeen, Aberdeen, UK

## Abstract

**Background:**

The UK Antimicrobial Registry (UKAR) was developed to capture data on real-world usage of recently launched antimicrobial agents.

**Methods:**

UKAR is an ongoing prospective registry of adult inpatients prescribed 11 eligible study drugs (cefiderocol, ceftaroline, ceftazidime/avibactam, ceftobiprole, ceftolozane/tazobactam, dalbavancin, delafloxacin, eravacycline, imipenem/cilastatin/relebactam, meropenem/vaborbactam and oritavancin). Data collected from participants’ medical records include demographics, infection site, comorbidities, microbiology isolates and susceptibility, treatment regimen and outcomes. Primary outcome is clinical resolution of infection measured 28 days post cessation of study drug.

**Results:**

In the first 20 months, 631 participants were recruited, 56% male, with a median age of 60 years. Overall, 44.8% of patients were treated for lower respiratory tract infection, 18.0% for systemic infections including sepsis and 11.1% for urinary tract infection. Comorbidities were common (>90%), 81% of participants had a documented history of resistant organism colonization and only a small proportion of patients received an eligible study drug while in critical care. For Gram-negative agents ceftazidime/avibactam, cefiderocol and ceftolozane/tazobactam predominated, and for Gram-positive agents 94% received dalbavancin. Empirical use was seen in 4.9% of Gram-negative and 66.2% of Gram-positive prescriptions. Where patient outcome was evaluable, infection resolution was seen in 69% and 64% of Gram-negative and Gram-positive participants, respectively.

**Conclusions:**

The UKAR provides real-world data on the use of novel antimicrobials confirming they are sometimes used empirically as well as for directed therapy to treat both complex and common infections, and often for multiresistant pathogens. The study is a novel and important resource to support the judicious use of these drugs.

## Introduction

High-quality data on how new antimicrobials are used in real-world clinical practice is needed to fully understand their effectiveness in complex and drug-resistant infections and in patients with significant comorbidity to help target their use. Such information is also important to support innovative approaches to funding, such as the NICE value-based subscription model,^1^ piloted from 2022 to support hospitals in England to utilize two antimicrobials, cefiderocol and ceftazidime/avibactam, for a fixed annual fee rather than paying the market cost per patient treatment course. Extension of this approach is underway to include all new antimicrobials licensed in the UK.

The UK Antimicrobial Registry (UKAR) was developed by the BSAC and researchers from the University of Aberdeen^2^ to collect data from hospital patients across the UK prescribed new antimicrobials, including the two in the NICE pilot. Similar registries in other countries have studied the use of specific antimicrobials,^3–9^ but UKAR^10^ is unique in that it aims to incorporate all newly licensed antimicrobials. UKAR will assess the effectiveness and safety of included antimicrobials across various clinical indications. The registry, which includes hospitals from all four UK nations, began recruiting participants in May 2023. Herein we share an overview of patients recruited to December 2024, to illustrate the breadth of information that the UKAR can provide that we feel is clinically and politically important.

## Methods

The UKAR protocol is available (https://osf.io/a3tu5/) but in brief: hospital inpatients aged ≥18 years were eligible for participation if prescribed any of the study drugs within the last 60 days, during their current stay in hospital. For this narrative overview, the 11 study drugs (new drugs available at inception of the study in 2021 and shown in Table 1) are separated into anti–Gram-positive and anti–Gram-negative agents, based on their target organisms and their deployment in clinical practice. Demographic information, and clinical and microbiological data were collected from participants’ medical records, including infection site, comorbidities, microbiology isolates and susceptibility, and treatment regimen. Index of deprivation was derived from participants’ postcode of residence.^10–13^ Data were collected and uploaded to an electronic case report form up to 28 days after the last administration of study drug, and 56 days after commencing treatment. Long-term follow-up data were collected 6 months and 12 months after the end of treatment.

**Table 1.** Baseline characteristics of UKAR study population

		Gram-negative^a^ (*N* = 261)		Gram-positive^a^ (*N* = 216)	
		*n*	(%)	*n*	(%)
**Demographic characteristics**					
Sex (male)		138	(53)	127	(59)
Median age (IQR), y		63	(46–72)	55	(42–71)
Ethnicity	White	210	(80.5)	196	(90.7)
	Mixed	2	(0.8)	3	(1.4)
	Asian or Asian British	29	(11.1)	—	
	Black or Black British	6	(2.3)	1	(0.5)
	Other ethnic groups	6	(2.3)	—	
	Not stated	8	(3.1)	16	(7.4)
Quintiles of deprivation	1 (least deprived)	56	(21.5)	52	(24.1)
	2	47	(18.0)	40	(18.5)
	3	47	(18.0)	37	(17.1)
	4	41	(15.7)	36	(16.7)
	5 (most deprived)	61	(23.4)	35	(16.2)
**Eligible study drugs**					
Ceftazidime/avibactam		109	(41.8)		
Cefiderocol		69	(26.4)		
Ceftolozane/tazobactam		61	(23.4)		
Imipenem/cilastatin/relebactam		16	(6.1)		
Meropenem/vaborbactam		6	(2.3)		
Eravacycline		—			
Dalbavancin				204	(94.4)
Ceftaroline				8	(3.7)
Ceftobiprole				3	(1.4)
Delafloxacin				1	(0.5)
Oritavancin				—	
**Type of infection**					
Lower respiratory tract infection		117	(44.8)	4	(1.9)
Systemic infections and conditions including sepsis		47	(18.0)	25	(11.6)
Urinary tract infection		29	(11.1)	—	
Intra-abdominal infections		25	(9.6)	6	(2.8)
Bone and joint infection		17	(6.5)	71	(32.9)
Skin and subcutaneous tissue infection		12	(4.6)	78	(36.1)
Infective endocarditis		1	(0.4)	18	(8.3)
Thoracic infection		—		4	(1.9)
Other		13^b^	(5.0)	10^c^	(4.6)
**Reasons given for initial choice of eligible study drug (multiple reasons could be selected^d^)**					
Susceptibility results (this episode)		152	(58.2)	47	(21.8)
Previous microbiology data (historical)		96	(36.8)	43	(19.9)
Risk of antimicrobial resistance		12	(4.6)	3	(1.4)
Renal impairment		3	(1.1)	1	(0.5)
No alternative		17	(6.5)	7	(3.2)
Allergy		8	(3.1)	9	(4.2)
Treatment failing		38	(14.6)	14	(6.5)
Facilitate OPAT		—		44	(20.4)
Facilitate discharge		2	(0.8)	51	(23.6)
Other		43	(16.5)	76	(35.2)

^a^Data are presented as *n* (%) unless otherwise stated.

^b^Including CNS infection (*n* = 1) and TB (*n* = 1) and 11 marked ‘other’.

^c^Including dental infection (*n* = 1) and 9 marked ‘other’.

^d^Reasons are not mutually exclusive therefore percentages sum to >100%.

Initial data were collected at recruitment (demographics, type of infection, eligible study drug, and reason for choice of study drug) but full data collection was not undertaken until the primary outcome—clinical resolution of infection, defined as resolved (no symptoms and clearance of infection), unresolved (infection still present) or relapsed (symptoms improved but the initial infection recurred within treatment period)—was reached. In this analysis, some information was only available on the sub-cohort in which outcome data were known.

Participant recruitment commenced in May 2023 and is ongoing. The database lock for the current analysis was 1 January 2025.

Ethical approval for the UKAR was received in England/Wales/Northern Ireland (ref: 23/LO/0018) and Scotland (ref: 22/SS/0006).

## Results

**Figure 1.**
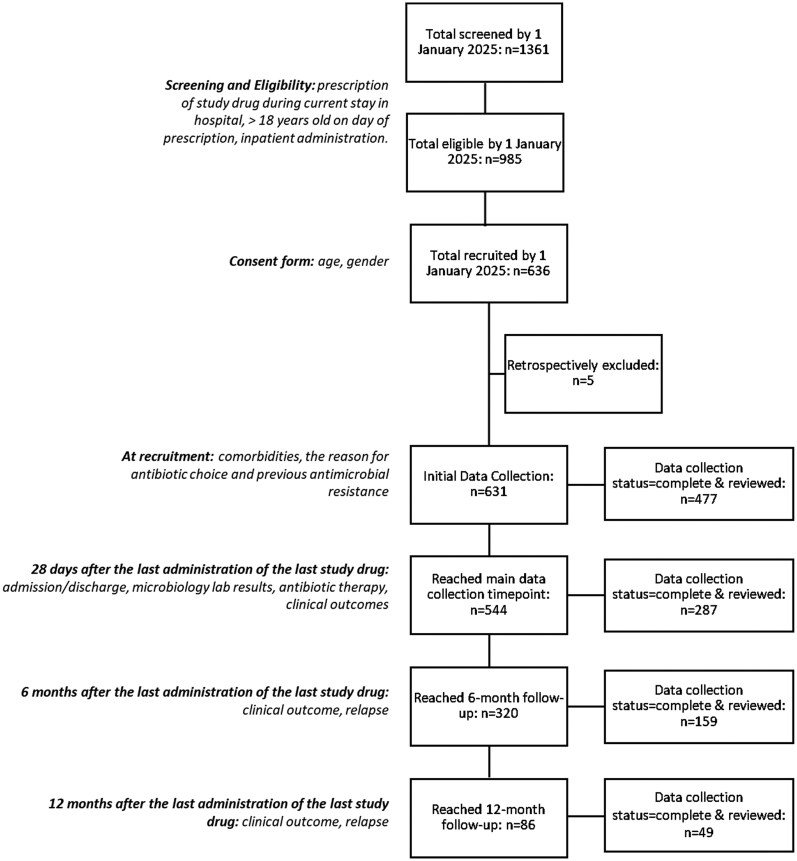
Flowchart of participant recruitment and data collection.

By 1 January 2025, 1361 patients from 45 hospitals had been prescribed a study drug and were screened for eligibility: 985 were eligible and 636 participants were recruited (Figure 1). Data are available on reasons for non-eligibility and will be presented elsewhere. Five participants were retrospectively excluded. Median age (IQR) was 60 (43–72) years and 56% were male. Comorbidities were common (>90% of participants), and most had a documented history of resistant organism colonization: 81% and 51% in Gram-negative and Gram-positive groups, respectively. Fifty-five percent of participants were treated for Gram-negative infection, and three agents predominated (ceftazidime/avibactam, cefiderocol and ceftolozane/tazobactam: 41.8%, 26.4% and 23.4%, respectively). The remaining 45% of participants were treated for Gram-positive infection, predominantly with dalbavancin (94%). A small proportion of patients received a study drug in critical care (15.5% of those prescribed Gram-negative agents and 2.1% receiving Gram-positive agents). Baseline demographic and clinical characteristics of the study population are shown in Table 1.

Gram-negative agents were mainly used for lower respiratory tract infection (44.8%). Systemic infections and conditions (including sepsis) (18.0%) and urinary tract infections (11.1%) were the next most common types of infection. For Gram-positive agents, most common infections treated were acute bacterial skin and skin structure infections (36.1%) and bone and joint infections (32.9%).

Fewer than 10% of participants overall received treatment in critical care settings despite having arrangements in place for consent of patients without capacity.

The most cited reason for the choice of drug was antimicrobial susceptibility (Gram-negative agents 58.2% and Gram-positive agents 21.8%). A significant proportion received a Gram-positive drug to facilitate discharge (23.6%) and a further 20.4% specifically to facilitate outpatient parenteral antimicrobial therapy (OPAT).

Data on suspected pathogens were available for 287 participants: 142 patients on Gram-negative agents, and 145 Gram-positive. Gram-negative agents were prescribed for *Pseudomonas aeruginosa* (39.4%), *Klebsiella pneumoniae* (19.0%) or *Escherichia coli* (9.2%) infections. In only seven instances (4.9%) was a Gram-negative agent prescribed without a suspected pathogen, whereas for participants prescribed Gram-positive agents no suspected pathogen was recorded in 33.8%. *Staphylococcus aureus* was the most common Gram-positive isolate found, in 45.8% of samples, with an MRSA rate of 11.4% in these samples. Study drugs were prescribed empirically for 31.0% and 25.5% of infections treated with Gram-negative and Gram-positive agents, respectively. For Gram-negative agents, other antibiotics were prescribed prior to the eligible study drug (59.2%) or concomitantly (66.2%), and 47.2% of patients received other antibiotics after study drug discontinuation. For Gram-positive agents, the majority of participants had prior antibiotic use (87.6%), but fewer received concomitant therapy or other antibiotics after study drug discontinuation (30.3% and 41.4%, respectively).

Clinical outcome data were available for 287 participants. Twenty-eight days after the end of treatment, 64.8% of Gram-negative infections were resolved, and in 15.5% the infection was unresolved or had relapsed. Twenty patients (14%) had died, and for 9 of these (45%) it was recorded that death was due to infection. For Gram-positive agents, 42.1% of infections had resolved and 20.7% of infections were unresolved or relapsed. Although few patients had died, in 33.8% of patients prescribed a Gram-positive agent the clinical outcome was non-evaluable.

In total 159 (33.3%) patients had reached the 6 month follow-up timepoint. Ten treated with Gram-negative agents, and two on Gram-positive agents had experienced relapse of infection (12.8% and 2.5%, respectively). At 12 months, the proportions were 11% and 5%, from 28 and 21 participants, respectively.

UKAR is also set up to capture adverse events but to date very few have been reported.

## Discussion

UKAR is the first prospective UK-wide antimicrobial registry designed to capture real-world use and outcomes from multiple newly licensed Gram-negative and Gram-positive agents. Over 600 patients were recruited from 45 hospitals during its first 20 months, with baseline clinical data complete for nearly 500. As expected, the majority of patients had comorbidities and a high proportion of patients had a history of resistant infection, particularly those with Gram-negative infections.

Combination therapy was commonly used, and detailed analyses of combinations used together with resistance mechanisms for Gram-negative isolates will be included in a future publication.

A key strength of UKAR is its prospective design with granular patient-level data on prescribing of WHO Watch/Reserve antimicrobials,^14^ including large specialist centres and district general hospitals across all four UK nations. Another strength is the electronic database system for the registry, which has the benefit of allowing all contributing parties to log in to record their entries and review their own data.

In terms of limitations, robust data collection is challenging due to variability in patient information systems and in the use of electronic prescriptions. Participation in UKAR is voluntary so not all hospitals in the UK are included. Staff capacity for obtaining patient consent and data collection is also a limitation, which means not all patients receiving study drugs are included. However, our screening data indicate that the majority of those patients prescribed a study drug fulfil eligibility criteria (72%) and recruitment of eligible patients is also high (65%). Thus, although we cannot rule out selection bias, the effect is likely to be small. The UKAR is designed as an inpatient registry, therefore some patients receiving eligible study drugs are excluded if treated as outpatients. For several included study drugs patient numbers are very low so it is difficult to draw any conclusions on their use. As with all studies, incomplete data can impact on full analyses of outcomes and regular communication with hospital teams helps to minimize this.

Looking to the future a virtual registry would allow data from all eligible patients to be captured. To explore this a pilot study has been completed to replicate the UKAR dataset utilizing the national data mart held by NHS National Services Scotland.

### Conclusions

This initial narrative review shows the breadth of data collected by the UKAR and its utility in answering important questions about effectiveness of included antimicrobials, which is important clinically and politically. With recruitment of hospital sites and study participants, continuing until at least May 2027, the power of the registry will further increase.

## Transparency declarations

J.S. works for BSAC on a consultancy basis and is Lead for the UKAR Programme. J.A.T.S. is a recent council member of BSAC, a member of the British Society for Allergy and Clinical Immunology Penicillin Allergy working party and a panel member of the ESCMID guideline for antibiotic allergy. J.A.T.S. has had research income from NIHR, EPSRC, MRC, Wellcome Trust, John Moulton Foundation and Lumix diagnostics; and has done consultancy work for Tillotts Pharma and Medtronic (2021) to design/deliver an educational event and consensus survey, respectively. I.B. reports investigator-initiated research funding from Shionogi BV for research on Gram-negative infections outside the submitted work; honoraria from Shionogi BV, Menarini UK and Tillotts Pharma; and participating in advisory boards for GSK and Eumedica. R.A.S. is President of BSAC, co-lead of the BSAC OPAT initiative, and Chair of the Scottish Antimicrobial Prescribing Group. R.A.S. has received fees for consultancy work for NAPP/Mundipharma, Pfizer and Advanz and for speaking at educational events for Menarini and Shionogi. R.P., J.W., R.I.R.M., G.J.M. and G.T.J. are employees of the University of Aberdeen, which has received a research grant award from the BSAC for the running of the UKAR. BSAC has received funds for this registry from Pfizer, Menarini and Shionogi. All remaining authors report no conflicts of interest.
